# Sexual and reproductive health service utilization and associated factors among youth workers at Kombolcha Industry Park, Kombolcha town, Northeast Ethiopia, 2023

**DOI:** 10.3389/fpubh.2024.1367421

**Published:** 2024-09-16

**Authors:** Addisu Endalew, Bereket Kefale, Natnael Atnafu Gebeyehu, Belete Birhan

**Affiliations:** ^1^Department of Public Health, College of Medicine and Health Science, Wollo University, Dessie, Ethiopia; ^2^Department of Reproductive and Family Health, Collage of Medicine and Health Sciences, Wollo University, Dessie, Ethiopia; ^3^Department of Midwifery, College of Medicine and Health Sciences, Wolaita Sodo University, Wolaita Sodo, Ethiopia; ^4^Department of Psychiatry, College of Medicine and Health Sciences, Wolayita Sodo University, Wolayita Sodo, Ethiopia

**Keywords:** sexual, reproductive, health, Industry Park, youth

## Abstract

**Background:**

Youth is a decisive age that shapes the direction of their life and that of their family. However, due to the host of biological, social, and economic factors, youth Industry Park workers can be at high risk of adverse sexual and reproductive health outcomes. Therefore, assessing youth sexual and reproductive health service utilization and associated factors among youth workers is very crucial for timely intervention to their gaps.

**Objective:**

This study aimed to assess sexual and reproductive health service utilization and associated factors among youth workers working at Kombolcha Industry Park Kombolcha, Northeast Ethiopia, 2023.

**Methods:**

An institutional-based cross-sectional study was conducted at Kombolcha Industry Park, Kombolcha, among a total of 422 youth workers from 1 January to 30 January 2023. A simple random sampling technique was used to access a total of 422 youth workers in Kombolcha Industry Park. The data were collected using pre-test, structured, and interviewer-administered questionnaires. Data were entered into Epi Data version 3.1 and were exported to SPSS version 26 for analysis. Bi-variable and multivariable binary logistic regression analyses were performed. Adjusted odds ratio with 95% confidence interval was estimated to measure the strength of the association. The level of statistical significance was declared at a *p* value of less than 0.05.

**Results:**

Overall utilization of reproductive health services was 45.50%. Being married [adjusted odds ratio (AOR) = 5.76, 95% CI (2.94, 11.25)], near distance of sexual and reproductive health (SRH) facility to home [AOR = 2.57, 95% CI (1.60, 4.14)], having good knowledge [AOR = 9.23 95%CI (4.88, 17.44)], and good attitude about SRH [AOR = 2.06 95% CI (1.29, 3.28)] were significantly associated with youth SRHs utilization.

**Conclusion:**

Youth sexual and reproductive health service utilization among youth workers of Kombolcha Industry Park was low. Ensuring SRHs availability and accessibility, empowering youth with knowledge of SRHs, and advocating sexual and reproductive health services to develop a good attitude might be important in improving reproductive health service utilization. Future researchers should address segments of the population other than Kombolcha Industry Park.

## Introduction

### Background

Sexual and reproductive health is a state of complete physical, emotional, mental, and social wellbeing, not just the absence of a disease or infirmity in all matters relating to sexuality and the reproductive system and its functions and processes (1, 2). According to the World Health Organization (WHO), youths are classified as young people aged 15–24 years (3). The Ethiopian National Youth Policy, on the other hand, targets young people aged 15–29 years (4).

There were 1.8 billion young people aged 10–24 years among which 1.2 billion youth aged 15–24 years globally in 2019, accounting for one out of every six people worldwide, in which Africa comprises 19% (above 226 million) of the global youth population (5). Young people make up the greatest proportion of the population in sub-Saharan Africa, with more than one-third of the population aged 10–24 years (6), and 33.8% of the total population in Ethiopia is between the ages of 10 and 24 years (7). Approximately, 85,000 people are currently employed at 13 industrial parks across Ethiopia and 95% of the workforce are youths, where 85% of them are young girls (8).

The Industry Park worker faces sexual and reproductive health concerns due to limited access and knowledge, leading to increased risks of sexually transmitted infections (STIs), HIV, and unwanted pregnancies (9, 10). Common global challenges, such as conservative socio-cultural norms and attitudes toward premarital sex and gender, significantly affect unmarried young people’s access to necessary information and services for a healthy adulthood transition (11). The World Health Organization estimates that 70% of premature deaths in adults are adolescent and youth behaviors. Unintended pregnancies account for 121 million pregnancies annually, with 60% ending in abortions and 45% being unsafe (12). Unsafe abortions, which hospitalize 7 million women annually and contribute to 5–13% of all maternal deaths, are one of the leading causes of maternal death (13). In 2020, 374 million new HIV infections were reported, with 1.75 million adolescents aged 10–19 years living with the virus, 70% of whom were girls, with 90% living in sub-Saharan Africa (14).

A study conducted among young women migrant workers working in the IZ Park in Hanoi, Vietnam showed that the overall utilization of sexual and reproductive health (SRH) among those who have sex among unmarried youth was 20.1%, and SRH utilization among married participants was 71.4%. Among those who used SRH services, the most likely services used for SRH were gynecological exams and counseling (15). In Eastern and Southern Africa, the region most affected by HIV, only 25% of girls and 17% of boys aged 15–24 years received the result of their most recent test (16). Contraceptive demand for youth is high almost everywhere, with an average of 84.7 and 80% for Latin American countries and sub-Saharan African countries, respectively. This implies that levels of unmet need for contraception are higher for sexually active youths, indicating specific problems with access to sexual and reproductive health rights (SRHR) for them. Thus, the unmet need for single adolescents is 24.4 and 41.4% on average in LAC and SSA, respectively (17).

Ethiopia’s youth-friendly sexual and reproductive health service utilization is 42.73% (18); however, low utilization of family planning services and high-unintended pregnancy and abortion complications occur. Factors associated with youth-friendly service utilization include grade levels, close-to-home services, male sex, discussing sexual activity with family and friends maternal education, age, reproductive issues, living arrangements, knowledge about sexual and reproductive health, and school involvement (18, 19).

It is evidenced that youths were visiting SRH services to receive SRH information for counseling services, to obtain a condom, a Voluntary Counseling and Testing (VCT), for treatment of STIs, and for post-abortion care (20). A study conducted in Ethiopia also showed that there was a 42.1% sexual risk behavior (21), 19% of youths reported having had premarital sexual intercourse with a mean age of 16.48 years at the first sexual intercourse (22), self-reported STI prevalence was 19.5% (23), and the abortion rate was also high in unmarried youths (24).

Different efforts and strategies are being applied to enhance the SRH health of youths, aligning with the Millennium Development Goal (MDG), Sustainable Development Goal (SDG 3, “Ensure healthy lives and promote well-being for all at all ages”) (25), and the WHO adolescent health strategy (12). Ethiopia is one of the countries that are attempting to incorporate different strategies to enhance youth SRH utilization (4), implement a minimum service package for adolescent and youth health at industry parks (8), and develop a national adolescent and youth reproductive health strategy (26, 27). To create job opportunities for youth and align with Sustainable Development Goals, the Ethiopian Government has invested in export-oriented manufacturing industrial parks. Many of these parks are now operational, providing jobs for tens of thousands, mainly young women with limited education who have migrated from rural areas to cities (28). Adolescence represents a critical transitional phase in life during which individuals are particularly susceptible to various health risks. These risks encompass sexual and reproductive health challenges, such as HIV/AIDS, unintended pregnancies, unsafe abortions, early marriages, childbearing, and STIs. Therefore, the study assesses SRH service utilization and associated factors among youth workers working at Kombolcha Industry Park.

## Materials and methods

### Study area

The study was conducted at Kombolcha Industry Park in Kombolcha town, Northeast Ethiopia. Kombolcha town is located 25 km from Dessie, 477 km from Bahir Dar, and 376 km from Addis Ababa. The Kombolcha Industrial Park, found in Kombolcha town and built in 2014 on 75 ha of land, has nine industrial sheds and will create jobs for 20,000 people. The Industrial Park is dedicated to apparel products and textiles (29). The total number of workers in Kombolcha Industrial Park: male = 955, female = 2,589, with a total of 3,544. Among the 3,544 people permanently working in Kombolcha Industry Park, 2,356 were youth. Every factory worker works according to the labor law recommendations (8 h per day) (30). The clinic operated by the Industry Park Development Corporation offers a range of services, including First Aid, and Ambulance Service, as well as health information and educational resources related to health.

#### Study design and period

- An institution-based quantitative cross-sectional study design was conducted from 1 January to 30 January.

### Population

#### Source population

- Youth working at Kombolcha Industry Park, in Kombolcha town were considered as source population.

#### Study population

- All youth workers who worked at Kombolcha Industry Park during the data collection period were considered as the study population.

#### Eligibility criteria

##### Inclusion criteria

- All youths aged 15–24 years and working at Kombolcha Industry Park in Kombolcha town for at least 1 year were included in the study.

##### Exclusion criteria

- Youths who had been working less than 1 year in the park and those who had been seriously ill throughout the study period were not included.

### Sample size determination

The sample size required for this study was calculated using a single population proportions formula considering the following assumptions. Since there is no study conducted on Industry Park workers’ utilization of SRH service, the prevalence was taken at 50%, with a 95% confidence level, 10% non-response rate, and a 5% margin of error.

Therefore, the sample size is given as follows:

$$n={\left(Z\;a/2\right)}^{2}\;p\left(1-p\right)/d^{2}$$

where,

*n* = the sample size.
(*Z a*/2)^2^ = at 95% confidence interval *Z* value (*a* = 0.05) = 1.96.
*p* = utilization of SRH were 50% (0.5).
*d* = margin of error at 5% (0.05).

= (1.96)^2^ (0.5) (1–0.5)/(0.05)^2^ = 384.

10% non-response rate = 10/100*384 = 38.

The minimum sample size is therefore 384 + 38 = 422.

### Sampling procedure and method

Initially, all youths working in Kombolcha Industry Park were listed by name and age, and then a simple random sampling technique was employed to select the study subjects from youths working in Kombolcha Industry Park. Youths were selected by using a simple random sampling technique from the register list.

#### Study variables

##### Dependent variable

Sexual and reproductive health service utilization: Yes/No.

##### Independent variables

Socio-demographic factors: age, sex, ethnicity, level of education, and housing condition.
Psychological and behavioral: participation in HIV AIDS club, discussion with peers and family about SRH, preferred sex of health provider, and youth knowledge and attitude toward SRHS.
Health-related factors: The provision of comprehensive care, privacy and confidentiality, the designated consultation duration, the operational hours of the health facility, the availability of recreational activities, and factors related to affordability and accessibility in terms of distance from one’s residence.
Workplace-based factors: availability of SRH club (including HIV/AIDS club), availability of clinic in the park, and availability of SRH education and information session.

#### Operational definition

Youth: According to the World Health Organization (WHO), youths are classified as young people aged 15–24 years (3).
SRH service utilization: It was measured by asking the respondent’s history of SRH utilization in the last 1 year. A respondent was considered as utilizing SRH services if he or she used one or more of the following SRH services (family planning, STI screening and treatment, HIV testing, and counseling safe abortion care) (20, 31).
The attitude of youths toward SRH services: This study used a five-point scale to assess unmarried youths’ attitudes toward SRH services. The mean score for each component was computed and dichotomized into good and poor on a Likert scale ranging from 1 (strongly disagree) to 5 (strongly agree). Respondents who scored above and equal to the mean were labeled as having a good attitude toward SRH service and those who scored below the mean were labeled as having a poor attitude toward SRH service (32).
Knowledge of SRH utilization: Four questions were used to measure the knowledge of the respondents on SRH utilization. When respondents got the right answer, it was coded as yes “1”; if not, it was coded as no “0” for the four questions. Respondents who scored above the mean value were regarded as having good knowledge, whereas those who scored below the mean value were considered as having poor knowledge about SRH health service utilization (32).
VCT service utilization: Youth who ever received HIV counseling and testing services in the last 12 months.
History of STIs and SRH problems: It comprises respondents’ report of the occurrence of any STIs in the last 1 year, unwanted pregnancy, and abortion. Respondents were asked questions such as “Have you had any STIs in the past 1 year?” and “Have you ever obtained unwanted pregnancy?” (31).
SRH service accessibility: The term “accessibility” was used in this study to refer to geographical accessibility as perceived by youngsters. Youths who reside within a 30-min walking distance of their house and health facility will be grouped as having good geographical accessibility and low accessibility otherwise (31).
Abortion care: It is care given to youth undergoing termination or end of a pregnancy in a health facility with minimal medical standards, either by induced or spontaneously before fetal viability, which is conventionally taken to be less than 28 weeks from the last normal menstrual period (33).
Unsafe abortion: It is terminating an unwanted pregnancy by self-medication or in a traditional way by traditional healers in an environment lacking medical standards (33).

### Data collection

Data collection was conducted utilizing a pre-tested structured questionnaire in Amharic, which was adapted from various sources (31, 34–37). The data-gathering process involved three data collectors, comprising two clinical nurses and one clinical midwife, all operating under the supervision of the principal investigator. The questionnaire encompassed a range of factors, including socio-demographic, behavioral, psychological, health system-related, and Industry Park-related elements.

### Data quality management

Structured questionnaires were first prepared in English and then translated into Amharic for data collection, and finally retranslated back into English in order to keep the consistency of the questions. The training was given to three data collectors and supervisors focusing on data collection techniques, the objectives of the study, and how to ensure the quality of data. Before data collection, pre-tests were conducted among 5% of the total sample size and tested in Woldiya agro-processing factory youth workers, and corrections were made before the actual data collection. The questionnaire demonstrated a Cronbach’s alpha of 0.74. The collected data were checked for completeness and clarity by the principal investigator daily. Data cleaning and cross-checking were also done before analysis.

### Data processing and analysis

After checking for its completeness, data obtained from each respondent were entered into epidata 3.1 versions and then exported to SPSS version 26 for analysis. Descriptive statistics analysis was employed. Mean, median, and percentage were used for describing different variables based on the nature of the variables, and the result was presented using tables and graphs. Bivariable binary logistic regression analysis was performed, and variables with a *p*-value of less than 0.25 were entered into multivariable logistic regression analysis. Finally, variables with a *p* value of less than 0.05 in a multivariable binary logistic regression analysis were considered statistically significant, and the adjusted odds ratio with its 95% confidence interval was considered to measure the strength and significance of the association.

### Ethical considerations

Ethical approval was obtained from the Ethical Review Committee of Wollo University College of Medicine and Health Science. A letter of cooperation was written to Industry Park. The purpose and importance of the research were explained to each study participant, and the data were collected after obtaining full informed written consent. Confidentiality of information was maintained by omitting any personal identifier from the questionnaires, assuring that their parents never had access to their responses and the data were stored in a safe place, where no one except the principal investigators had access. The participants were informed of their right to participate or deny participation at any stage and non-participation would not affect them in any way.

## Results

### Socio-demographic characteristics

A total of 422 youths were enrolled in the study with a response rate of 100%. The mean (±) age of the respondents was 21.95 ± 1.56 years old, and 369 (87.40%) of the respondents were within the age group of 20–24 years. Out of the participants, 316 (74.90%) were women, and 342 (81.00%) were currently single. Out of all participants, 326 (77.30%) were Muslim religion, and 96(22.70%) were Orthodox Christian. Regarding youth’s educational status, 272 (64.00%) were in secondary and preparatory schools (Table 1).

**Table 1:** Socio-demographic characteristics of youth working in Kombolcha Industry Park, Northeast Ethiopia, 2023.

Variable	Category	Frequency	Percentage
Age	20–24	369	87.40
	15–19	53	12.60
Sex	Male	106	25.10
	Female	316	74.90
Marital status	Single	342	81.00
	Married	80	19.00
Religion	Muslim	326	77.30
	Orthodox	96	22.70
Educational status	Primary	77	18.20
	Secondary	272	64.50
	College and above	73	17.30

### Health service accessibility and behavioral characteristics of respondents

In total, 303 (71%) and 5 (1.10%) of the respondents know at least one and four SRH service components, respectively. A total of 365 participants have information on SRH services and 336 (79.60%) participants were aware of at least one health facility where SRH services could be delivered. Government health institutions, youth-friendly service centers, private health facilities, and NGO health facilities were SRH services delivery points as cited by 336 (79.60%), 152 (36.00%), 35 (8.29%), and 16 (3.79%) of the respondents, respectively. In total, 353 (83.60%) youths preferred weekend time as the most favorable service hour, and 58 (13.70%) preferred holidays for service hours. The majority of the respondents, 269 (69.90%), have ever had sex, 301 (71.30%) respondents have good knowledge of SRHs, and 221 (52.40%) respondents have a favorable attitude toward the SRH services (Table 2).

**Table 2:** Health service accessibility and behavioral characteristics of respondents (*N* = 422).

Health facility accessibility	Frequency	Percentage
Confidentiality of health facility for secret use of SRH	Yes = 147	34.80
	No = 275	65.20
Distance of health faculty from home <30 min of walk	Yes = 168	38.90
	No = 254	61.10
Preferred working hours		
Early in the morning	3	0.70
Afternoon	8	1.90
Weekend	353	83.70
In the holidays	58	13.70
Ever had sexual intercourse	Yes = 295	69.00
	No = 127	31.00
Perceived risk of SRH problem	Yes = 160	37.90
	No = 262	62.10
Knowledge about youth SRH	Good knowledge = 301	71.30
	Poor knowledge = 121	28.70
Attitude toward youth SRH	Good attitude = 221	52.40
	Poor attitude = 201	47.60

Among respondents knowledgeable about sexual and reproductive health services (SRHS), information sources included peers 146 (40%), mass media 106 (29%), anti-HIV/AIDS clubs 70 (19.5%), and health institutions 43 (11.5%).

### Utilization of SRH services

**Figure 1:**
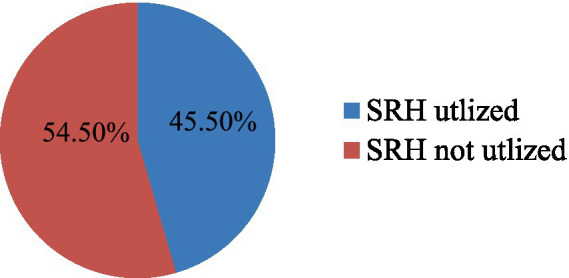
SRH service utilization of youths working in Kombolcha Industry Park Northeast Ethiopia (*n* = 422).

This study result showed that 192 (45.50%), 95% CI (40.3–50.70) of overall study subjects had received at least one component of the SRH services in the last 12 months (Figure 1).

The largest portion of the participants, comprising 97 individuals (50.50%), reported that they had obtained sexual and reproductive health (SRH) services from youth-friendly service centers. A significant number, 77 individuals (40.00%), indicated that they had received these services from government health facilities. Additionally, 54 participants (28.10%) reported obtaining SRH services from the Family Guidance Association of Ethiopia, while 45 individuals (23.40%) stated that they had received these services from IPDC clinics.

**Figure 2:**
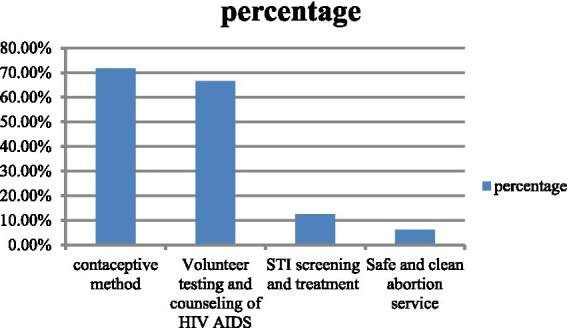
Component of SRH utilized by youth workers working in Kombolcha Industry Park (*n* = 192).

Contraceptive methods were the most commonly used SRH services, with 138 individuals (71.80%) using this service, followed by Volunteer Test and Counseling for HIV/AIDS with 128 individuals (66.60%). Additionally, STI screening and treatment were used by 24 individuals (12.50%), while safe and clean abortion services were used by 12 individuals (6.25%) (Figure 2).

The result also showed that 264 (26.70%) youths who had sex in the last 12 months had used contraceptive methods. In this study, 230 (54.50%) of the study participants did not use SRH services in the last 12 months. The reasons for not using SRH services were due to fear of being seen by family (78, 34.00%), the distance to the health facility (71, 31.30%), respondents did not know where SRH service is provided (71, 31.10%), and due to high cost of the service (8, 3.50%).

### Factors associated with the utilization of SRH services

Bi-variable and multivariable binary logistic regression analyses were performed to identify factors associated with sexual and reproductive health service utilization among youth Industry Park workers. Variables with a *p* value of less than 0.25 in the bi-variable binary logistic regression analysis were entered into multivariable binary logistic regression analysis. Variables eligible for multivariable analysis include age, sex, marital status, perceived risk of SRH problem, knowledge of SRHs, and attitude toward youths’ SRHs. In the final multivariable binary logistic regression analysis, the marital status of the youths, nearness of the SRHS facility to the participants’ home, knowledge of SRH, and favorable attitude toward youths’ SRH were identified to be significantly associated with youths’ SRH utilization at a *p* value of less than 0.05.

The odds of sexual and reproductive health service utilization among married youth workers were 5.7 times higher than among single youth workers [AOR = 5.76, 95% CI (2.95, 11.22)]. Youths with an SRHS facility near their home (less than 30 min of walk away) were 2.5 times more likely to utilize the SRH service than those with an SRHS facility far from their home [AOR = 2.57, 95% CI (1.59, 4.14)].

The odds of sexual and reproductive health service utilization among youth workers having good knowledge of SRHs were 9.2 times higher than youths having poor knowledge of SRHs [AOR = 9.23, 95%CI (4.88, 17.44)]. The odds of sexual and reproductive health service utilization among youth workers having good attitudes toward youth SRHs were two times higher than youths having poor attitudes toward SRHs [AOR = 2.06, 95%CI (1.29, 3.28)] (Table 3).

**Table 3:** Multivariable logistic regression analysis on factors associated with utilization of youth RH services among youth workers working in Kombolcha Industry Park 2023 (*N* = 422).

Variable	Category	SRHs utilization		COR, (95%CI)	AOR, (95%CI)
		Yes (%)	No (%)		
Age	20–24	175 (91.1)	194 (84.3)	1.91 (1.036, 3.52)	1.75 (0.79, 3.66)
	15–19	17 (8.9)	36 (15.7)	1	1
Sex	Male	37 (19.3)	69 (30)	1	1
	Female	155 (80.7)	161 (70)	1.79 (1.13, 2.83)	1.56 (0.90, 2.66)
Marital status	Single	131 (68.2)	211 (91.7)	1	1
	Married	61 (31.8)	19 (8.3)	5.17 (2.95, 9.04)	5.76 (2.94, 11.25)**
Perceived risk of SRH problem	Yes	88 (45.8)	72 (31.3)	1.85 (1.24, 2.76)	1.53 (0.96, 2.44)
	No	104 (54.2)	158 (68.7)	1	1
Distance of health facility from their home	< 30 min	99 (51.6)	65 (28.3)	2.7 (1.81, 4.04)	2.57 (1.60, 4.14)**
	>30 min	93 (48.4)	165 (71.7)	1	1
Knowledge of SRHs	Good knowledge	173 (90.1)	128 (55.7)	7.25 (4.22, 12.4)	9.23 (4.88, 17.44)**
	Poor knowledge	19 (9.9)	102 (44.3)	1	1
Attitude about SRH	Good attitude	113 (58.9)	108 (47)	1.61 (1.1, 2.37)	2.06 (1.29, 3.28)**
	Poor attitude	79 (41.1)	122 (53)	1	1

**Variables significantly associated with SRHs utilization at *p* value < 0.01. COR, Crude odds ratio. AOR, Adjusted odds ratio.

## Discussion

This study was aimed at assessing the sexual and reproductive health service/SRHs/utilization and associated factors among youth workers working at Kombolcha Industry Park. This institution-based cross-sectional study revealed that the utilization of at least one SRH service in the last 12 months was 45.5% [95% CI: 40.3–50.7]. Being married, distances of health facility from home, knowledge of respondents on SRHs, and attitude toward SRHs utilization were significantly associated with SRH service utilization. This study was in line with the study conducted on the pooled prevalence of youth-friendly sexual and reproductive health services utilization and its determinants in Ethiopia, which is 42.37% (18), and the study conducted in Awabel District, 41% (38), Bale Zone Goba town (39), and Nigeria (40). This result is higher than that of the study conducted in Woreta, 24.6% (35); Debertabour, 28.8% (41); Nekemte, 21.2% (42); Hadiya zone (38.5%) (34); and Addis Ababa bole sub city, 33.5% (31). However, the finding of the study is lower than the study conducted in the West Arsi zone, 58.6% (20) and Harer town, 64% (43). This difference might be due to the participant’s socio-demographic and socio-economic characteristics, target population difference, definition of SRH service utilization, and the availability and accessibility of SRH service.

The most frequently utilized SRH service was the contraceptive method, 138 (71.8%), which is almost similar finding to that of the study conducted in Gondar town (44) and Nigeria (40). This finding is higher than that of the study conducted in Worta (35), Debertabour (41), and Deber Berhan town (36). This discrepancy might be due to socio-demographic variations due to their age, maturation, and freedom to take contraceptives. This study shows that volunteer test and counseling for HIV/AIDS, 128 (66.6%), are higher than the study conducted in Debre tabor (41), Bahir dar followed by STI screening and treatment in 24 (12.5%) and safe and clean abortion service in 12 (6.25%).

This study result revealed that married youth Industry Park workers more frequently used SRH services than those who were single. This finding was similar to the study conducted in Deber Tabor (32). The possible justification may be that married women have cultural freedom to utilize family planning to build families and hold activities as per their plan; they can freely discuss with their partner, families, and friends, which creates a good opportunity for adequate awareness and positive attitude toward SRH service needs.

Youth whose SRH facility was close to home were 2.57 times more likely to utilize SRHs than those whose SRH facility was far from home. This is similar to the study conducted at Woreta town (35). This is because health facilities are readily available and accessible in the surrounding area, allowing youths to use them conveniently and gain access to RH education and enabling them to utilize the service at whatever time they feel comfortable taking SRHs.

Participants who had good knowledge of youth SRHs were 9.23 times more likely to utilize SRH services than those who had poor knowledge. This finding is in line with the study conducted in East Gojjam and Bahir Dar (45). This might be due to the fact that youth workers with better knowledge and understanding of sexual and reproductive health issues might have better decision-making skills for reproductive health service utilization.

Participants who had a good attitude toward youth SRH service utilization were 2.06 times more likely to utilize SRH services than those who had a poor attitude toward youth SRHs. This study is similar to that of the study conducted in Bahir Dar. This is due to the fact that youth workers having a favorable attitude would motivate them to use reproductive service utilization.

The result of this study shows that there was low utilization of SRH services in the last 12 months among youth workers compared with other studies. This was worsened by perceived barriers such as fear of being seen by family, long distance from a health facility, and inconvenience of service hours of health facility because most youth workers’ preferred time is weekend SRH service. This implies that a lot has to be done on awareness creation about the nature of youth SRHS and to support the youth so that they can pay due attention to improve SRH services and make health facilities available to them that provide the services available for users at any time including weekends. Furthermore, the government and other stakeholders should strengthen youth awareness creation strategies and empower them to have good knowledge and positive attitudes toward youth SRH services utilization.

## Limitations of the study

Because this study examines personal and sensitive issues, obtaining honest responses about their sexual history from youth workers might have been difficult. These data might be prone to respondent bias. This study was based on cross-sectional data, which implies that the direction of causal relationships cannot always be determined. The quantitative study design did not allow for probing into certain areas that needed further qualitative description. The study was conducted in only one of the Ethiopian Industry Park, which means the findings may not be generalizable to Ethiopian Industry Park youth workers.

## Conclusion

This study showed that Kombolcha Industry Park youth workers’ SRH service utilization was low. This might make youth workers prone to different problems related to reproductive health, which, in turn, can increase the work dropout rate that affects the productivity of Industry Park, and which ultimately affects the country’s economy at large.

Being married, health facility close to home SRH service, having good knowledge of SRH service and its components, where to get the service, and having a good attitude about youth SRHs were significantly associated with youth SRH services utilization. This result highlights the need for governmental, non-governmental, and other stakeholders to develop an effective intervention in implementing youth-friendly SRH service utilization and promoting the service to reduce SRH-related problems.

## Recommendation

### To the Ministry of Health

The Federal Ministry of Health should pay attention and priority to their policymaking and program designing for the availability of SRH service delivery points to their working area for youths working in Industry Park.

### To the Industry Park Development Corporation

Industry Park Development Corporation of Ethiopia should open SRH service delivery points with well-equipped materials and advanced health professionals with the knowledge and understanding of youth’s character and IPDCs principle of working toward its youth workers that educate the youth to increase their knowledge and attitude about youths sexual and reproductive health service utilization.

### To Wollo University

Wollo University should work with IPDC on further research on SRH service utilization of Kombolcha Industry Park youth workers and give refreshment training for youths working in Kombolcha Industry Park to increase knowledge of SRH and to build a positive attitude about SRH.

### To the Kombolcha Health Department

The Kombolcha Health Department aims to make SRH facilities accessible to all youth, particularly those working in the Kombolcha Industry Park, by ensuring convenience and promoting more service centers.

## Data Availability

The original contributions presented in the study are included in the article/supplementary material, further inquiries can be directed to the corresponding author.
